# Supplementary material for: An ion-electronic hybrid artificial neuron with a widely tunable frequency
Source: Nat Commun. 2025 Aug 25;16:7911. doi: 10.1038/s41467-025-63195-7 (PMC12379150; doi:10.1038/s41467-025-63195-7)
Supplement: Supplementary file 1 — Supplementary Information [file 41467_2025_63195_MOESM1_ESM.pdf]

Supplementary Information for

An ion-electronic hybrid artificial neuron with a widely tunable frequency

Jidong Li[1,2], Wei Zhao[1,3], Chenwei Fu[1,3], Zhenpeng Zhai[1,3], Pengfei Xu[3], Xinyuan Diao[1], Wanlin Guo[1,2], Jun Yin[1,2]*

[1]State Key Laboratory of Mechanics and Control for Aerospace Structures, Key Laboratory for Intelligent Nano Materials and Devices of the Ministry of Education, Nanjing University of Aeronautics and Astronautics, Nanjing, 210016, P. R. China.

[2]Institute for Frontier Science, Nanjing University of Aeronautics and Astronautics, Nanjing, 210016, P. R. China.

[3]College of Aerospace Engineering, Nanjing University of Aeronautics and Astronautics, Nanjing, 210016, P. R. China.

*To whom correspondence should be addressed: yinjun@nuaa.edu.cn

This PDF file includes:

Supplementary Notes 1-5

Supplementary Figures 1-23

Supplementary Table 1

Supplementary References

Supplementary Note 1: Analysis of load impedance effect on firing activity of hybrid neurons

The successful activation of the memristor's threshold switching is crucial for the firing behavior of our hybrid neuron. To analyze the impact of load impedance on this activation process, we first consider the quasi-steady I_m-V_m characteristic of an isolated memristor, obtained by gradually sweeping the current. As depicted in Fig. 1c, a negative differential resistance (NDR) region emerges.

As the input voltage (V_{in}) is swept from 0 V, the operating point (V_m, I_m) moves along the high-resistance branch of the *I-V* curve (green solid line), as shown in Supplementary Fig. 2b. Upon reaching the threshold voltage ($\mathrm{V_{th}}$), a rapid transition occurs from point $\mathrm{P_{thL}}$ to $\mathrm{P_{thH}}$, followed by a shift to the low-resistance branch (blue solid line) as V_{in} continues to increase. When the input voltage is swept back, the operating point traverses the low-resistance branch until it reaches the hold voltage ($\mathrm{V_h}$) at the point $\mathrm{P_{hH}}$. A further decrease in V_{in} triggers a sudden drop to $\mathrm{P_{hL}}$, and the operating point returns to the high-resistance branch. This abrupt resistance changes at $\mathrm{V_{th}}$ and $\mathrm{V_h}$ underlies the threshold switching behavior of our memristor.

However, when a load resistor (R) is connected in series with the memristor (Supplementary Fig. 2a), the load line, representing the voltage-current relationship of the circuit, becomes inclined with a slope of $-1/\mathrm{R}$ due to Ohm's law:

$$I_m = -\frac{1}{\mathrm{R}}V_m + \frac{V_{in}}{\mathrm{R}} \tag{S1}$$

For very low load resistances, the load line sweeps up and back, exhibiting threshold switching behavior in a similar manner mentioned above when the load line is tangent to the memristor's I_m-V_m curve (Supplementary Fig. 2c). In contrast, for high load resistances with slopes lower than the minimum slope in the NDR region, the operating point (V_m, I_m) continuously traverses the NDR region, eliminating the threshold switching behavior (Supplementary Fig. 2d). Consequently, spike generation is constrained by the following condition on the load impedance to ensure the switching behavior of memristor:

$$R < R_{max} \tag{S2}$$

Furthermore, to model the solid-liquid interface of hybrid neurons, we must consider an electrical double layer (EDL) capacitor (C) parallel to the faradic load resistor R. The charge and discharge dynamics of this capacitor introduce non-equilibrium effects, enabling dynamic switching between the high-resistance and low-resistance states of the memristor even under constant DC input voltage V_{in}.

To maintain this non-equilibrium state and ensure spike generation, the memristor voltage V_m must satisfy specific conditions. When the memristor is in the high-resistance state $\mathrm{R_H}$, the voltage V_m across the memristor must exceed the threshold voltage $\mathrm{V_{th}}$ to trigger the transition to the low-resistance state before the capacitor fully discharges. Conversely, when the memristor is in the low-resistance state $\mathrm{R_L}$, V_m must be less than the holding voltage $\mathrm{V_h}$ to return to the high-resistance state. Consequently, the load resistance R must satisfy the following conditions:

$$\frac{V_{in}}{R + \mathrm{R_H}} \mathrm{R_H} > \mathrm{V_{th}} \tag{S3}$$

$$\frac{V_{in}}{R + \mathrm{R_L}} \mathrm{R_L} < \mathrm{V_h} \tag{S4}$$

These can be further simplified to:

$$R < \frac{\mathrm{R_H}}{\mathrm{V_{th}}} V_{in} - \mathrm{R_H} \tag{S5}$$

$$R > \frac{\mathrm{R_L}}{\mathrm{V_h}} V_{in} - \mathrm{R_L} \tag{S6}$$

Combined with the constraint from Eq. S2, these conditions define the permissible relationship between R and V_{in}, which is confined to the green region of the R-V_{in} map, as depicted in Supplementary Fig. 10. This theoretical analysis aligns well with the experimentally observed boundaries in Fig. 2f.

While this analysis simplifies the memristor as an ideal two-state device and the solid-liquid interface as a constant R-C parallel circuit, it neglects the complex nonlinearities arising from thermodynamic switching, electrochemical reactions, and dynamic coupling with the EDL capacitor during charge and discharge processes. Nevertheless, the analysis provides valuable insights into the fundamental behavior of hybrid neurons and aids in tuning their spiking modes experimentally.

Supplementary Note 2: Narrow frequency tunability in solid-state artificial neurons

A typical solid-state artificial neuron comprises a memristor with an RC circuit (Supplementary Fig. 5a). DC input voltage V_{in} is applied to stimulate continuous firing activity. As V_{in} decreases, the mean firing rate decreases as well. However, more and more significant fluctuations in the firing rate are observed as V_{in} approaches the threshold for firing activity as shown in Supplementary Fig. 5b. This instability limits its stable firing rate in a narrow window.

The RC circuit introduces a time constant that governs the charging and discharging behavior of the circuit, thereby controlling the firing rates. Analysis of the RC component's response to step voltage inputs reveals an exponential decay with a time constant that is independent of the input voltage (Supplementary Fig. 5c). Its response to a sum of individual input voltage is equal to the sum of the individual responses, indicating linear behavior of the charging and discharging process. This linear behavior and exponential decay of the RC circuit limits the firing frequency range of the solid-state neuron as illustrated in details below.

Before each spike, the memristor is in the high-resistance state, and its voltage exponentially decays toward an equilibrium state due to the discharging behavior of the RC circuit as shown in Supplementary Fig. 6. The memristor voltage v_m can be described by the following equation:

$$v_m = V_{eq} - (V_{eq} - V_0)e^{-\frac{t}{\tau}} \tag{S7}$$

where $\mathrm{V_{eq}}$ is the equilibrium voltage, $\mathrm{V_0}$ is the initial voltage, t is time, and τ is the time constant. When v_m reaches the threshold voltage $\mathrm{V_{th}}$, the memristor switches to a low-resistance state and a spike is initiated. The time (T) taken to reach $\mathrm{V_{th}}$ from $\mathrm{V_0}$ corresponds to the spiking interval. Any perturbation (δv_m) near the threshold voltage, potentially arising from thermal fluctuations or noise, will significantly impact the spiking interval (δt):

$$\delta v_m = \frac{(\mathrm{V_{eq}} - \mathrm{V_0})e^{-T/\tau}}{\tau}\delta t \tag{S8}$$

Therefore,

$$\delta t = \frac{\tau \mathrm{e}^{T/\tau}}{\mathrm{V_{eq}} - \mathrm{V_0}}\delta v_m \tag{S9}$$

When the spiking interval (T) is significantly larger than the time constant (τ), even small perturbations near the threshold voltage can lead to substantial fluctuations in the spiking interval. This exponential dependence of δt on T results in significant firing rate instability at low firing frequencies, as observed in Supplementary Fig. 5. Consequently, the tunable range of firing rates in solid-state neurons is typically limited in a single order of magnitude of the time constant. In contrast,

our hybrid neurons exhibit stable modulation over a wide range, from 0.06 Hz to 2.8 kHz, due to the varied time constant resulting from the nonlinear behavior of the solid-liquid interface, as shown in Fig. 2.

Supplementary Note 3: Mass transfer controlled electrochemical interface in the hybrid neuron

Electrochemical reactions at different electrode interfaces proceed at varying rates, resulting in different currents or impedances under the same voltage drive. The impedance corresponding to the electrochemical reaction originates from two aspects: charge transfer and mass transfer, which can be represented by the charge transfer resistance (R_{ct}) and the Warburg impedance (Z_w), respectively. R_{ct} inversely reflects the intrinsic ability of the electrode to transfer charge across the interface. However, even if R_{ct} was zero, the reaction current would not be infinite. In such a scenario, reactants at the electrode surface would be instantaneously consumed, and the reaction rate would become limited by the rate at which reactants can be replenished from the bulk solution. This replenishment occurs through mass transport mechanisms including concentration gradient diffusion, convection (e.g., induced by stirring), and migration driven by electric potential gradients. This transport is described by the Nernst-Planck equation:

$$\boldsymbol{J} = -D\boldsymbol{\nabla} c + c\boldsymbol{v} - \frac{Dze}{k_B T} c\boldsymbol{\nabla}\phi \qquad (S10)$$

where $\boldsymbol{J}$ is the mass transfer flux, $\boldsymbol{\nabla} c$ is the ionic concentration gradient, $\boldsymbol{v}$ is the flow velocity, $\boldsymbol{\nabla}\phi$ is the electric potential gradient, D is the diffusivity of the chemical species, z is the valence of ionic species, e is the elementary charge, k_B is the Boltzmann constant, and T is the absolute temperature. In this mass-transfer-limited regime, the rate of ion transport dictates the reaction rate and current, and this limitation is quantified by Z_w.

Electrochemical impedance spectroscopy (EIS) was performed on the liquid part of our hybrid neuron (Supplementary Fig. 9), involving redox reaction $Fe^{3+} + e^- \leftrightharpoons Fe^{2+}$ at electrode interface. It reveals the interplay between charge transfer and mass transfer processes. The equivalent circuit of the electrochemical interface, derived from EIS fitting, incorporates a Warburg impedance element to accurately model the observed behavior. At high frequencies (large ω), the impedance spectrum exhibits a semi-circular shape, characteristic of RC circuit dominated by the electric double layer capacitance and R_{ct}. In this high-frequency regime, the time scale is too short for significant mass transfer to occur, rendering Z_w negligible. Conversely, at low frequencies (ω approaching zero), the impedance spectrum exhibits a diagonal line shape, characteristic of Warburg diffusion dominated by the mass transfer. Thus, the high-frequency region is controlled by charge transfer, while the low-frequency region is controlled by mass transfer.

The operating frequency of our hybrid neuron (typically below several kHz) falls within this mass-transfer-controlled low-frequency regime. Within this regime, the electrochemical behavior is

determined by the voltage division across the redox solution, exhibiting the observed nonlinearity (Supplementary Fig. 8 and Fig. 2). Consequently, the time constant of the system can be tuned across several orders of magnitude by varying the input voltage, enabling wide frequency tunability without introducing substantial fluctuations (Eq. S9).

However, the upper limit of our HAN's firing rate is indeed primarily constrained by the ionic component, specifically the charging and discharging time constant (τ) of the electric double layer (EDL). This time constant is determined by the product of the interfacial faradic resistance (R_t) and the EDL capacitance (C_{dl}), given by $\tau = R_t \times C_{dl} = \rho \times c$, where ρ and c are the faradic resistance and the EDL capacitance per unit area. Thus, to enable faster operation, ρ and c need to be reduced. For instance, by replacing the ITO electrodes with platinum (Pt) electrodes, we successfully increased the spiking rate from approximately 5 kHz to ~20 kHz. This improvement is attributed to the faster electrode reaction rate of Pt, which significantly decreases ρ. However, further extending the firing rate for extremely high-throughput applications remains a challenge. Potential approaches for future work include increasing the faradic current density through the employment of catalytic layers, optimizing electrolyte composition for faster ion reactivity, or exploring alternative electrochemical systems with intrinsically lower time constants.

Supplementary Note 4: Configuration of the electrochemical elements for afferent sensing

The hybrid neuron's load resistance, determined by the electrochemical impedance of the redox solution, is directly related to the charge transfer rate at the electrodes $Fe^{3+} + e^{-} \rightleftharpoons Fe^{2+}$, which is sensitive to factors such as temperature, ionic concentration, and fluid flow velocity. This impedance sensitivity, which significantly influences firing behavior (Supplementary Note 1), enables the hybrid neuron to function as a sensory element, eliminating the need for separate external sensors.

The electrodes for the sensory cells were fabricated using low-resistance ITO films (1-5 Ω/square) coated on glass substrates. The ITO patterns were defined by laser etching (Foster Fiber Laser Marking Machine). A ~100 μm thick patterned PDMS film was sandwiched between the patterned ITO glass and another glass after plasma treatment and pressure assembly, forming a glass/PDMS/glass structure. This created a microcell for the redox solution. For specific applications, modifications were made to this basic structure (Supplementary Fig. 12 and Fig. 4).

For mechanosensation, the glass/PDMS/glass structure was modified with two holes drilled in the top glass for fluid inlet and outlet, creating a microfluidic channel. An Elveflow OB1 infusion system with an MFS D5 flow sensor precisely controlled the flow velocity of 0.5M Fe^{2+}/Fe^{3+} redox solution. An ultrasonic cleaning machine (80 W, 40 kHz) was employed to stimulate the mechanosensory hybrid neuron with vibration.

For chemosensation, a PDMS/glass configuration was created by combining a PDMS film with two holes to a glass. Droplets of high (1.8 M) and low (0.4 M) ion concentration solutions were placed in these holes, with two ITO electrodes exposed to the low-concentration solution. Initially, the low ion concentration prevented neuron activation. Subsequently, the two droplets were bridged via a glass capillary tube, with the high-concentration end positioned slightly higher to facilitate gravity-assisted diffusion of ions into the low-concentration solution. As the ionic concentration in the sensing region gradually increased, the neuron began to fire, with the firing rate increasing over time until saturation.

For thermosensation, a modified intravenous line drip setup delivered hot water droplets as heat stimulus sources. A thin and large glass cover (~500 μm thick) with patterned ITO electrodes was placed on top of the PDMS/glass structure to sever as the "skin" and prevent the hot droplets from contacting and diluting the redox solution (0.25 M).

For handwriting recognition, a 3×2 array of ITO electrodes was patterned on the top glass, creating a sensory interface for six thermosensory neurons sharing one electrochemical cell of 0.4M

Fe^{2+}/Fe^{3+} concentration. The bottom ITO glass served as a common ground. The thin top glass facilitated rapid heat transfer from finger contact to the underlying ITO electrodes. Finger pressure on the sensing pad, following the writing path of digits, stimulated corresponding hybrid neuron firing.

These modifications to the basic glass/PDMS/glass hybrid neuron architecture demonstrate its versatility as a platform for developing bioinspired sensors capable of detecting diverse external stimuli.

Supplementary Note 5: Spike detection and DBSCAN clustering

Spike detection and DBSCAN clustering were performed using custom Python scripts on waveforms acquired by the oscilloscope. Spikes were initially identified by detecting rising and falling edges, and extracting parameters including spike occurrence time, width, and amplitude. Subsequently, a Density-Based Spatial Clustering of Applications with Noise (DBSCAN) algorithm was employed to cluster the detected spikes based on their frequency and amplitude characteristics. To improve cluster separation, a weight factor was applied to the logarithm of the firing rate before performing DBSCAN. Finally, the DBSCAN algorithm assigned each spike to a cluster. The results were visualized by labeling spike points in the frequency-amplitude plot and each spike's peak point in the waveform plot with the corresponding cluster color.

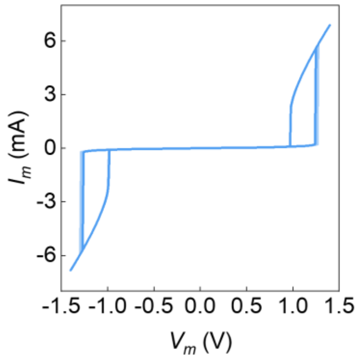

Supplementary Fig. 1. Bidirectional *I-V* characteristic of the Nb/NbTi$_x$O$_y$ threshold memristors under 100 cycles of symmetrical voltage sweeping.

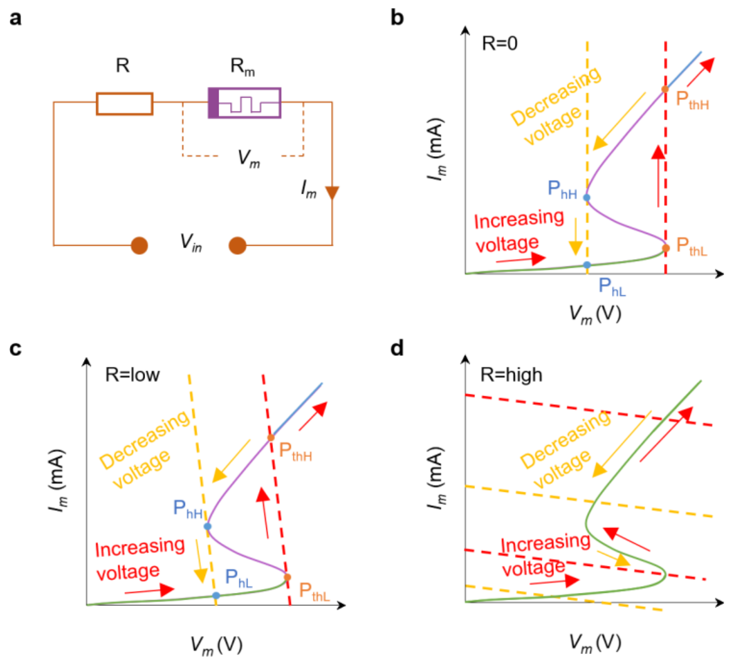

Supplementary Fig. 2. Load resistance constraints for memristor threshold switching. a) Circuit model used to analyze the influence of load resistance (*R*) on the memristor switching behavior. b-d) *I-V* curves of the memristor under three load conditions: b) zero load, c) low load, and d) high load. The red and yellow dashed lines represent the load lines during increasing and decreasing input voltage, respectively. P$_{thL}$ and P$_{thH}$ represent the threshold points switching from high-resistance state to low-resistance state. P$_{hH}$ and P$_{hL}$ represent the hold points switching from low-resistance state to high-resistance state. High load resistance suppresses threshold switching of memristor.

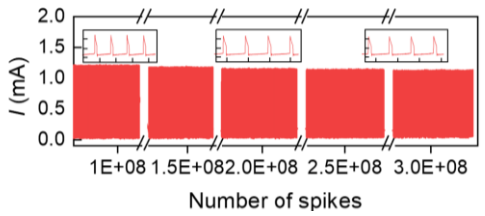

Supplementary Fig. 3. Long-term stability of HAN firing. The spike characteristics remain stable after 3×10^8 firing events.

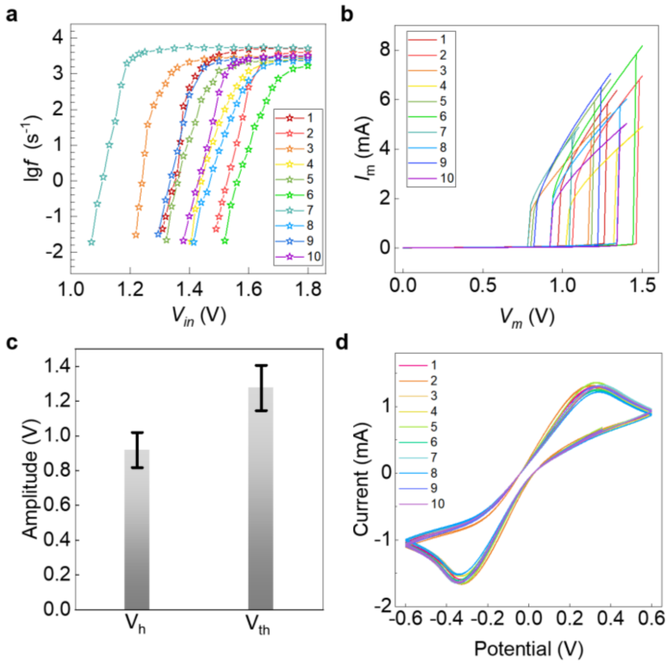

Supplementary Fig. 4. Device-to-device variability of the HAN. a) Log dependence of the firing frequency f on V_{in} of 10 HANs. b) *I-V* characteristics of the 10 HANs' memristor part, exhibiting obvious device-to-device variation. c) Statistical variation of the threshold voltage (V_{th}) and holding voltage (V_h) of the 10 memristors. d) Cyclic Volumetry of the 10 HANs' electrochemical cell part, exhibiting small variation.

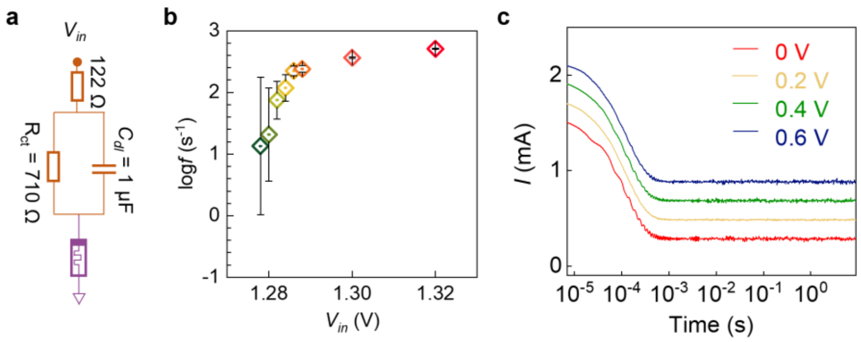

Supplementary Fig. 5. Firing frequency tunability of a solid-state electronic artificial neuron. a) Circuit of the solid-state artificial neuron based on a RC circuit connected in series with a memristor. b) Firing rate versus input voltage, demonstrating a narrow tuning window limited by the significant frequency fluctuations at low input voltages. Error bars represent the standard deviation (SD). c) Chronoamperometry of the RC circuit (brown part in a) at different input voltage (0V, 0.2V, 0.4V and 0.6V), demonstrating the same time constant independent of input voltage.

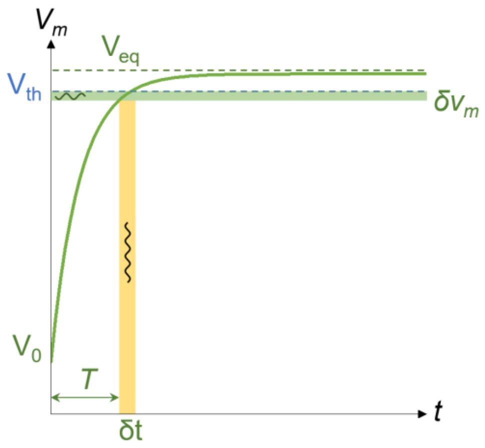

Supplementary Fig. 6. Causes of limitations of firing rate tuning in the solid-state artificial neuron. Illustration of the relationship between resting potential (V_{eq}), threshold voltage (V_{th}), and spike frequency (*t*) for the artificial neuron discussed in Supplementary Fig. 5. As the resting potential approaches the threshold voltage at low input voltages, small fluctuations in the threshold voltage (δV_m, illustrated by the green shaded region) can induce significant fluctuations in the spike frequency (δt, illustrated by the yellow shaded region), leading to instability of the firing rate.

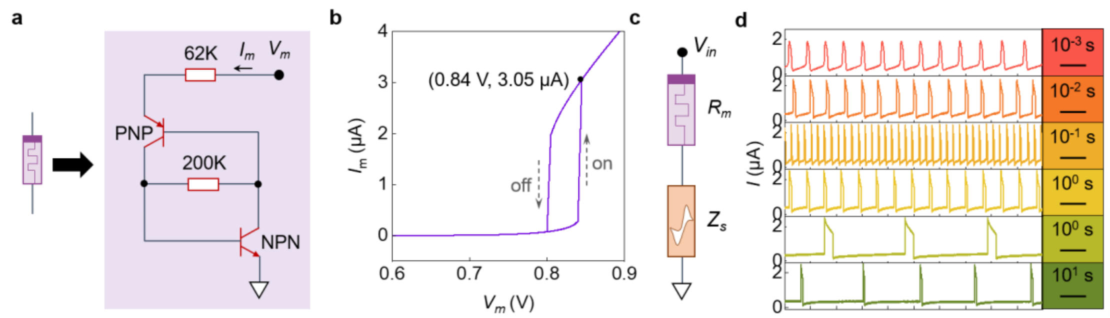

Supplementary Fig. 7. Transistor-based HAN exhibiting widely tunable firing frequency and low power consumption. a) Circuit schematic of transistor-based artificial neuron unit consisting of PNP and NPN transistors and two resistors. b) I_m-V_m characteristics of the transistor-based neuron exhibiting threshold switching behavior, similar to Nb/NbTi_xO_y memristor but with a much lower on current of ~3 μA. c) Schematic circuit of a HAN, composed of transistor-based neuron (purple element) in serials with a liquid cell (brown element). The working electrode is a Pt electrode with an area of only 100 square micrometers. d) Spike waveforms corresponding to different frequency.

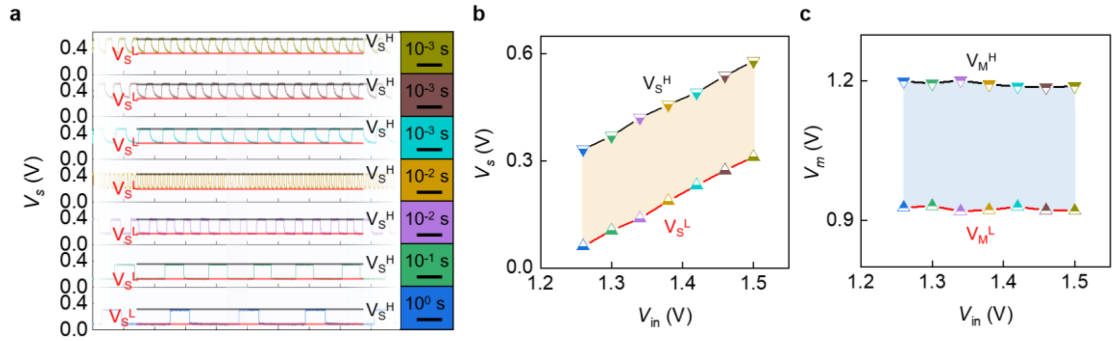

Supplementary Fig. 8. Voltage response across the solution during firing activity of hybrid neurons. a) Voltage waveforms across the solution during firing activity at different rates, highlighting the minimum (V_S^L) and maximum (V_S^H) voltages. b) Voltage range across the redox solution (V_s) as a function of input voltage V_{in}. V_S^H and V_S^L represent the highest and lowest voltage divisions across the redox solution during firing. c) Memristor voltage range (V_m) as a function of V_{in} during spiking. V_M^H and V_M^L represent the highest and lowest voltage divisions across the memristor during firing, respectively. Note that V_M^H and V_M^L remain nearly constant and are independent of V_{in}, corresponding to the intrinsic threshold voltage (V_{th}) and holding voltage (V_h) of the memristor.

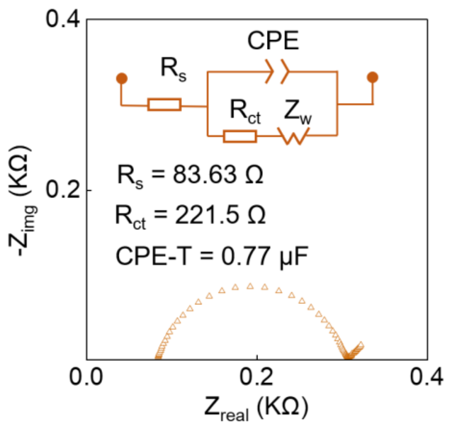

Supplementary Fig. 9. Electrochemical impedance spectrum of the Fe^{2+}/Fe^{3+} redox solution. The spectrum was measured using a two-electrode setup. The inset shows the equivalent circuit of the impedance spectrum, revealing an obvious Warburg impedance and mass transfer controlled electrochemical kinetics.

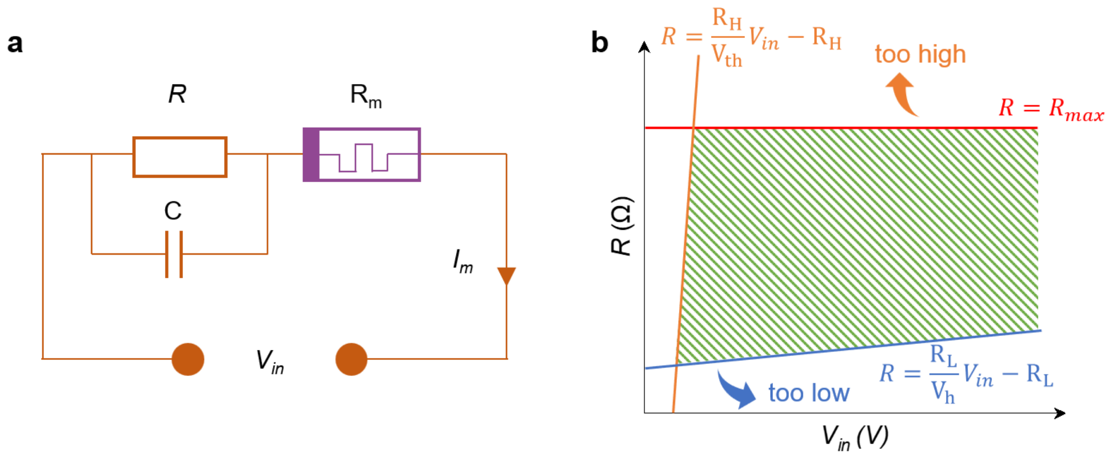

Supplementary Fig. 10. Constraint relationship between load resistance and input voltage for spike generation. a) Circuit model diagram for spike generation. b) Constraint relationship between load resistance (R) and input voltage (V_{in}). R-V_{in} with magnitudes only within the shaded diagonal region can spike generation be allowed.

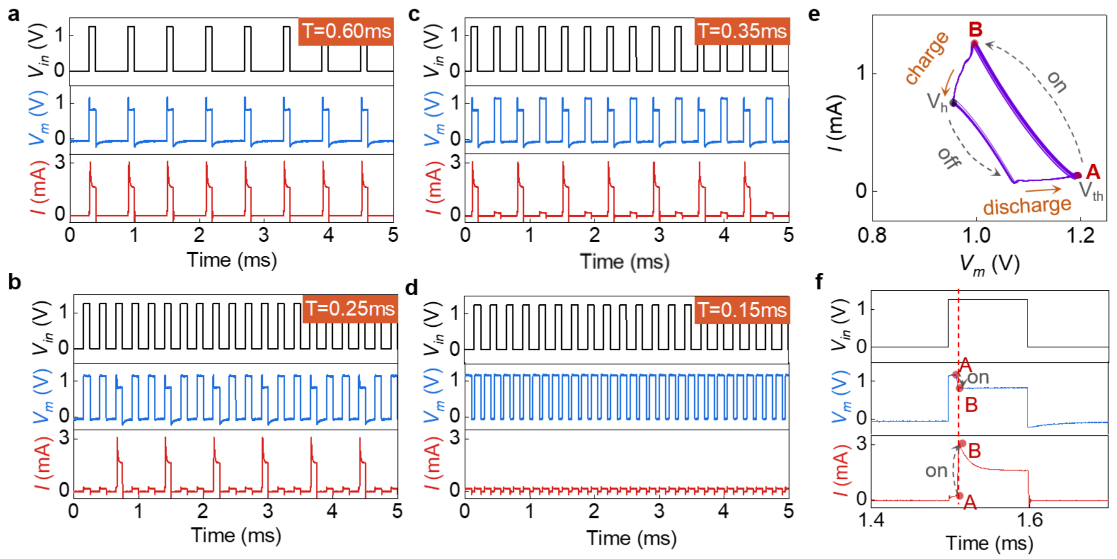

Supplementary Fig. 11. Spiking behaviour and hyperpolarization under varying stimulus rates. Input stimulus voltage waveforms (V_{in}), corresponding current spike waveforms (I), and memristor voltage responses (V_m) under different stimulus pulse intervals of (a) 0.60 ms, (b) 0.25 ms, (c) 0.35 ms, and (d) 0.15 ms. The input voltage pulses had a height of 1.25 V and a fixed width of 0.1 ms. Each input pulse stimulus is shown to induce hyperpolarization following the spike. e) Dynamic *I-V* characteristic of the memristor during firing activity, originally presented in Fig. 1f in the main text. It highlights the switch-on routine labeled as on from point A to B, contributing to the observed initial overshoot of I and V_m during each spiking event. f) Magnified waveform of V_{in}, V_m, and I during a typical spiking event. Gray dashed arrow labeled as on corresponds to the transient switch-on process in (e). Bandwidth of the current preamplifier (SR 570) employed for I acquisition is 1.0 MHz.

When an input pulse is applied, the memristor is initially in its high-resistance state, possessing an impedance significantly greater than that of the liquid component. Consequently, it instantaneously divides the majority of the input voltage, resulting in a distinct memristor voltage (V_m) pulse (approximately 1.17 V). If a spike is not elicited, the memristor simply maintains this high-resistance state, and V_m retains a high voltage division dictated by the input pulse voltage. This explains the observation of a V_m pulse without a concomitant current spike.

Conversely, if a spike is elicited, the memristor undergoes a rapid transition from the high-resistance (off) state to the low-resistance (on) state. This abrupt switch in resistance corresponds to the switch-on routine along the load line as shown in (e) and Supplementary Fig. 2c. As a result, a sharp and substantial increase in current (I) occurs, accompanied by a sudden drop in V_m from point A to B, which manifests as the initial overshoot observed during each spiking event as shown in (f).

Following this initial rapid drop, V_m approaches the holding voltage (V_h). However, the high differential resistance of the memristor in the vicinity of V_h induces significant decrease in current I while inducing only minor variations in V_m as shown in (e). Consequently, V_m exhibits an ultraslow decay, appearing to stabilize after the initial overshoot, before eventually returning to its resting state upon termination of the input pulse.

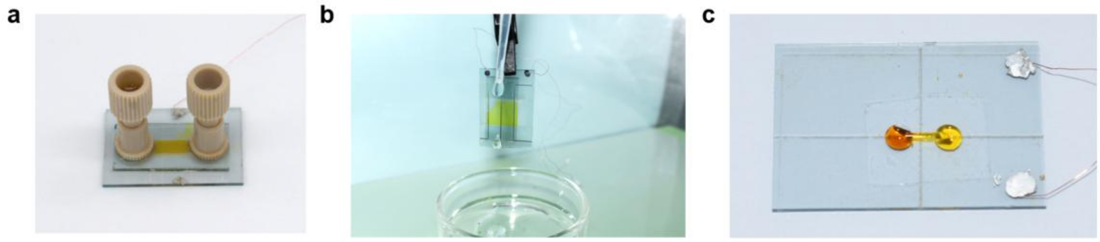

Supplementary Fig. 12. Optical images of hybrid neuron sensory cells. a) Microfluidic channel cell for flow sensing. b) Microchamber cell for temperature sensing. c) Chemosensory cell for sensing ionic diffusion between high- and low-concentration droplets.

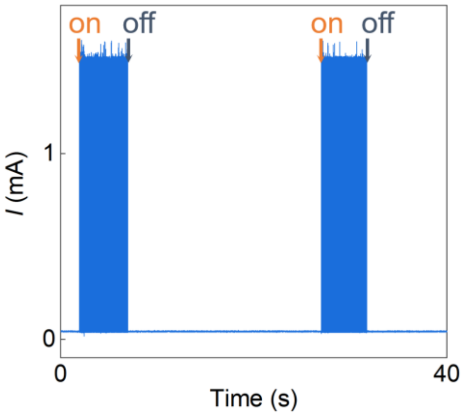

Supplementary Fig. 13. Response of hybrid neuron to ultrasonic vibration. The hybrid neuron, subjected to ultrasonic stimuli, exhibits rapid on/off firing patterns in response to the on/off cycles of the ultrasonic source (80 W, 40 kHz), demonstrating its sensitivity to ultrasonic vibrations.

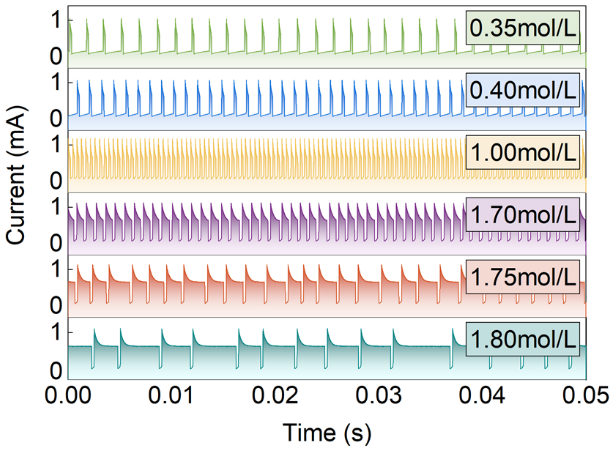

Supplementary Fig. 14. Effect of ion concentration on spike waveforms of HAN. Due to the short duration of the low-resistance state, the high impedance produced by the low ion concentration results in a narrower spike waveform. The low impedance produced by the high ion concentration results in a wider spike waveform due to the longer duration of the low-resistance state.

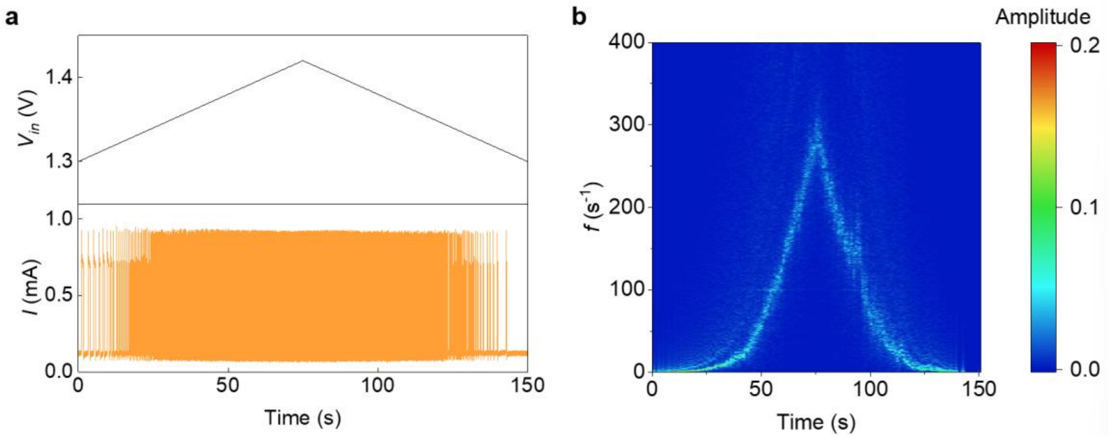

Supplementary Fig. 15. Frequency encoding of HAN for cockroach leg stimulation. a) Firing activity of HAN in response to linearly sweeping input voltage. b) Short-time Fourier Transform (STFT) of the HAN's current response. The firing rate increases with increasing input voltage, demonstrating the ability of the HAN to encode input voltage into a wide range of firing frequencies.

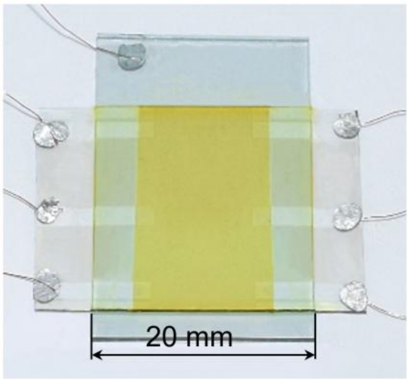

Supplementary Fig. 16. Optical image of the thermosensory array for handwritten digit recognition. A piece of ITO glass is patterned into a 3x2 array that serves as the sensing interface for six separate HANs. The yellow area represents the area filled with redox solution. The patterned ITO glass was placed facing upwards during the sensing process. Thicknesses of the bottom and top ITO glass are 0.5 mm. Thickness of the central electrochemical cell is ~0.1 mm.

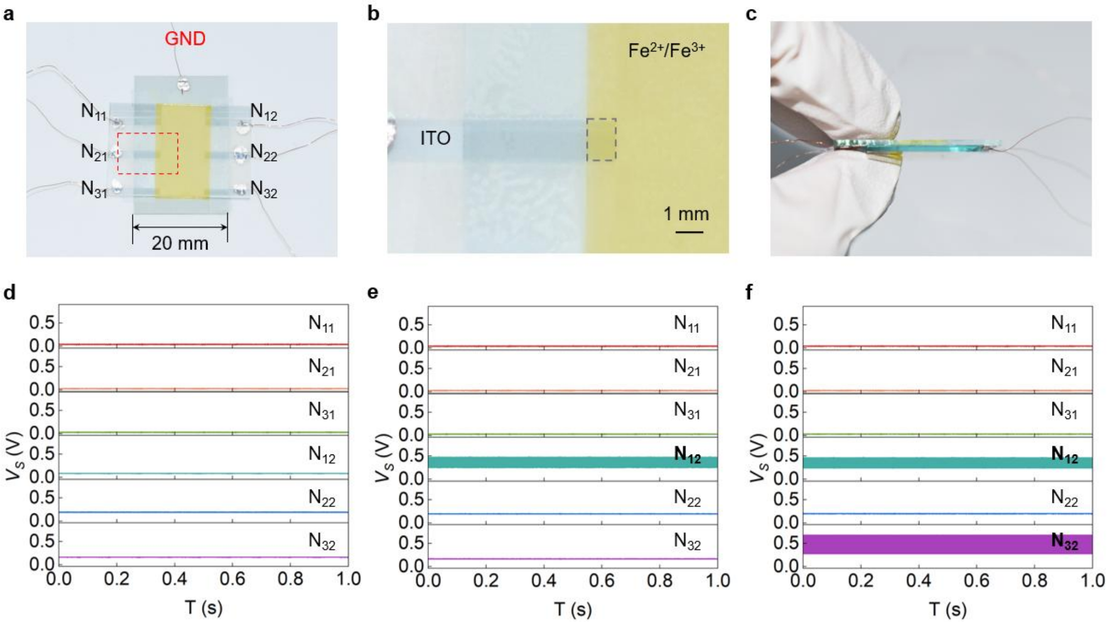

Supplementary Fig. 17. Independent operation of the HAN array. a) Optical top view of the HAN array, illustrating six individually wired working electrodes (N_{ij}, where i = 1,2,3 and j = 1,2) and a shared ground electrode (GND). The yellow region indicates the Fe^{2+}/Fe^{3+} redox solution, with the same area of the shared ground electrode. b) Magnified image corresponding to the red dashed box in (a), highlighting the working area by the gray dashed box. c) Optical side view of the HAN array, showing the approximate thicknesses of the bottom ITO glass (~1.1 mm), top ITO glass (~1.1 mm), and the central electrochemical cell (~0.1 mm). d) State of six resting HAN neurons at a critical voltage poised for activation. e) Activation of HAN N_{12} by increasing its input voltage, demonstrating no activation in other neurons. f) Subsequent activation of HAN N_{32} by increasing

its input voltage, confirming continued independent operation of HAN N_{12} (spiking at ~0.6 kHz) and absence of interfere in other neurons.

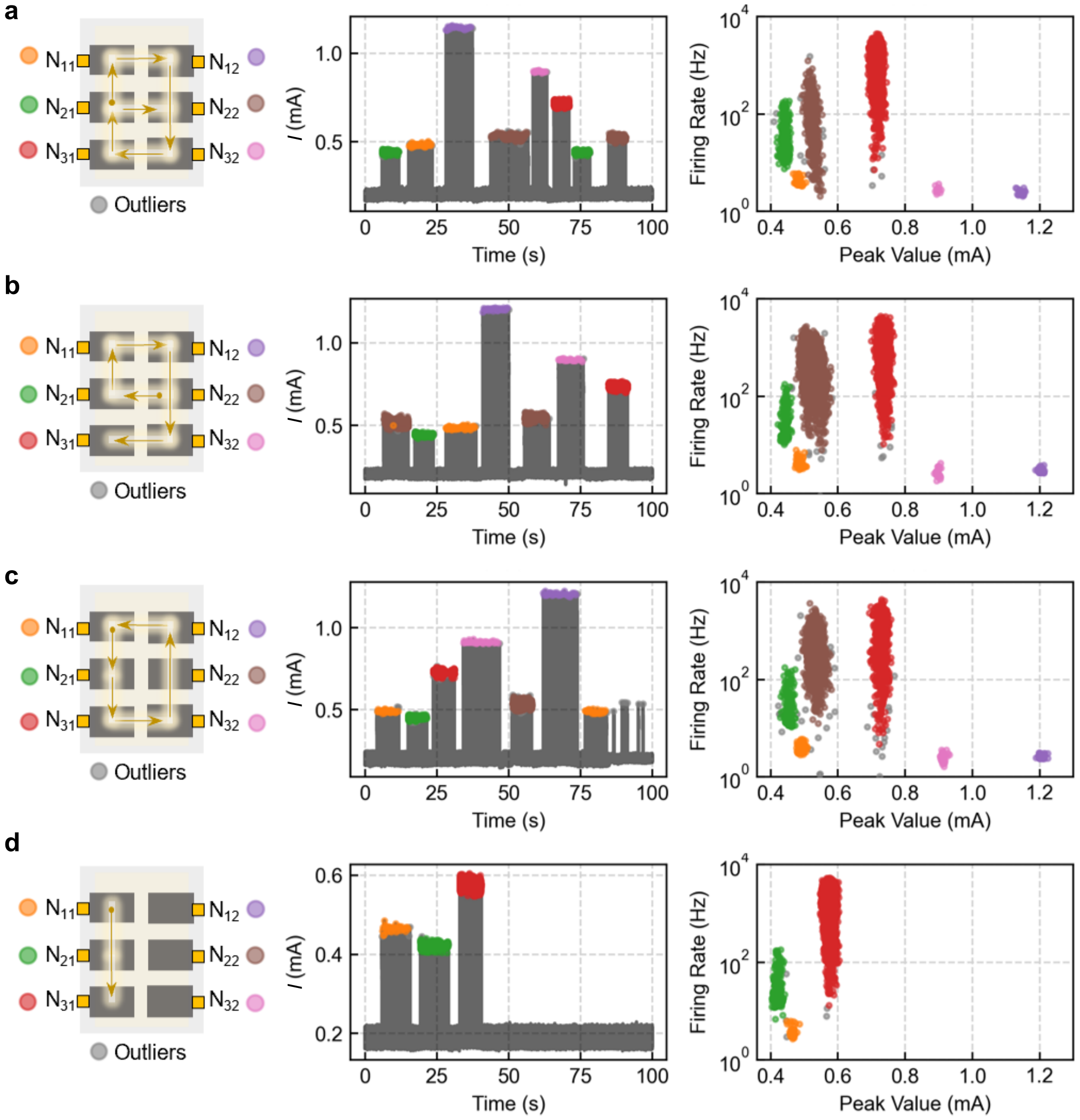

Supplementary Fig. 18. Typical digital handwriting trajectory recognition by DBSCAN clustering of 3×2 hybrid neuron signals. a-d) Trajectory and cluster analysis of handwritten digits 8, 9, 0, and 1. Left panels illustrate the handwritten digit trajectory with circular color labels representing the activation of specific neurons in the 3x2 array. Middle panels display the corresponding summed spike trains from the hybrid neuron array, with color-labeled spike peaks according to their DBSCAN cluster assignments. Right panels show DBSCAN results based on fire rate and spike amplitude characteristics.

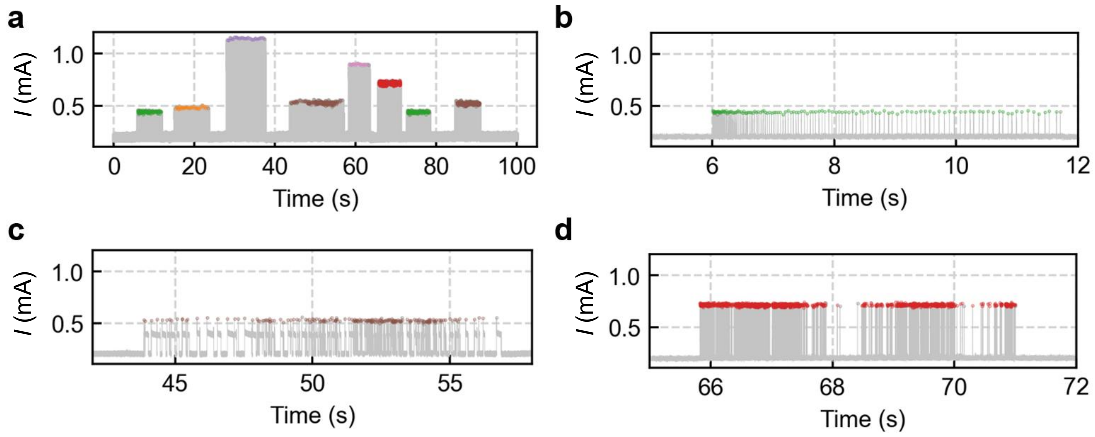

Supplementary Fig. 19. a) Output waveform of the sensing array in response to the handwriting of 8. b-d), Magnified spike waveforms extracted from a) corresponding to electrodes N_{21}, N_{22} and N_{31}, respectively, illustrating their broader firing rate range.

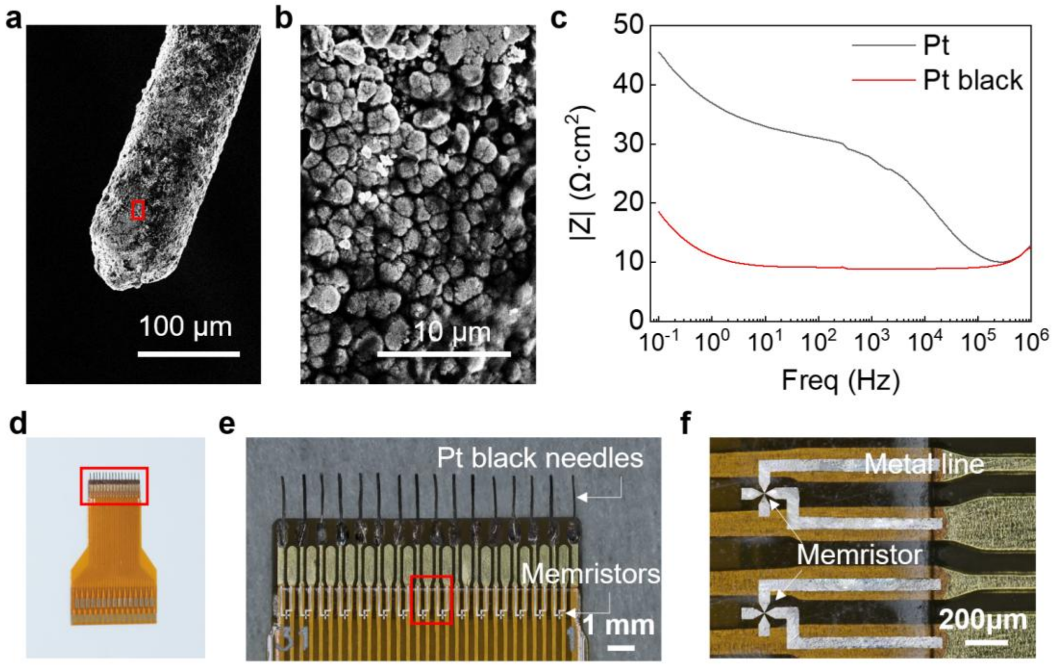

Supplementary Fig. 20. Characterization of platinum black (Pt-black) electrodes. a) SEM image of Pt wire electroplated Pt-black. b) Magnified SEM image corresponding to the red box region in (a). c) Bode plot of electrochemical impedance. The plot illustrates a substantial decrease in the electrochemical impedance for the Pt-black electrode compared to a bare Pt electrode of the same nominal footprint, particularly within the low-frequency region where the impedance is dominated by the faradic resistance. This reduction in impedance is result from the enhanced specific surface area of the Pt-black electrode. d) Optical image of flexible printed circuits (FPC) integrated with Pt-black needles (~100 µm in diameter) and $Nb/NbTi_xO_y$ memristor for HANs. e) Magnified image corresponding to the red box region in (d). f) Magnified image corresponding to the red box in (e).

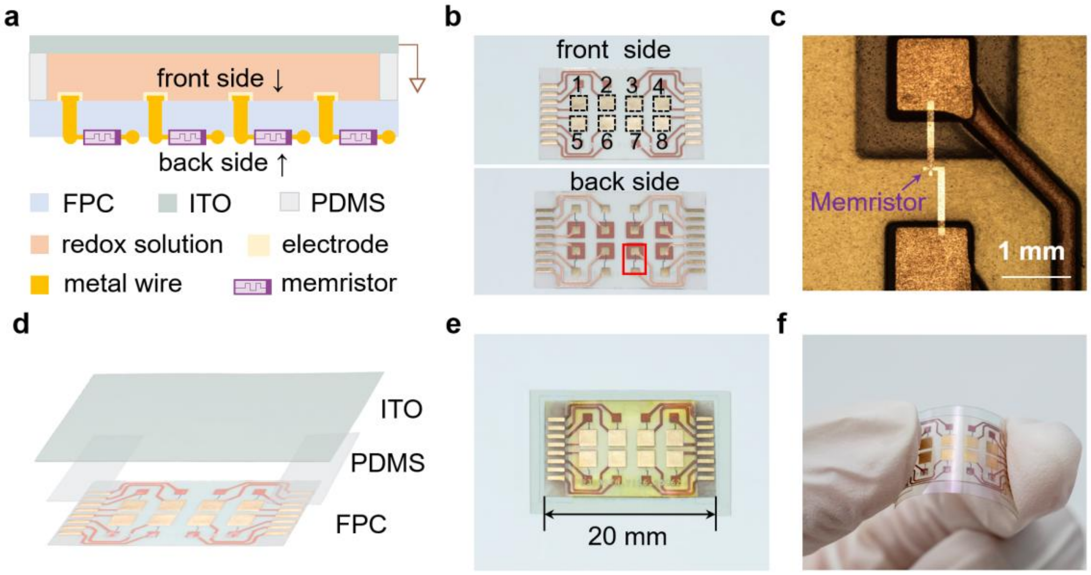

Supplementary Fig. 21. Strategies for three-dimensional integration of HANs. a) Schematic illustration of a proposed 3D integration scheme for HANs. An insulating plate serves as the Flexible Printed Circuit (FPC) substrate, with the solid-state memristor and electrical wiring integrated on its backside, while the electrochemical electrode is exposed on its frontside. b) Optical image of the FPC layer utilizing a transparent polyimide film as the insulating plate. Only 8 Au working electrodes are exposed on the front side of the FPC for the electrochemical cells as highlighted by the black dashed box. c) Magnified image corresponding to the red box region in (b), highlighting the memristor integrated on the back side of FPC. d) Schematic illustrating the assembly of the FPC-integrated HAN array device. e) Optical image of FPC-integrated HAN array. f) Optical image of the flexibly integrated HAN array.

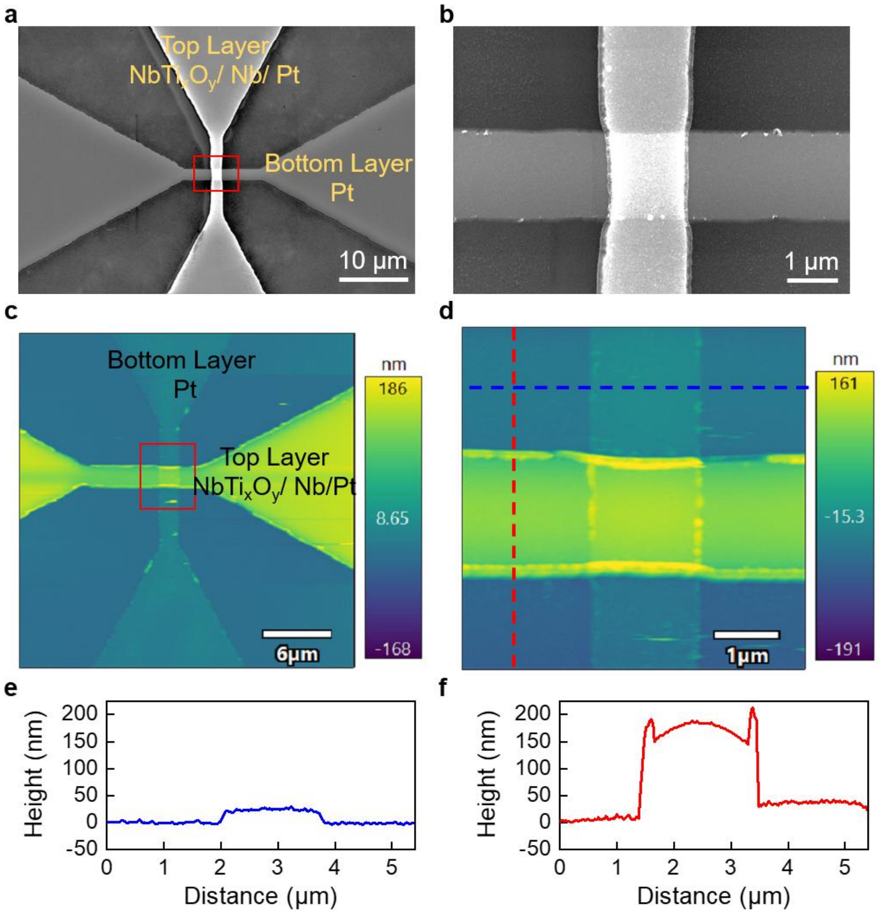

Supplementary Fig. 22. Morphological characterization of the threshold memristor. a) SEM image showing the crossbar configuration of the memristor device. b) Magnified SEM image corresponding to the red dashed box area in (a). c) AFM image depicting the height morphology of the memristor. d) Magnified AFM image corresponding to the red dashed box area in (c). e) Relative height profile corresponding to the blue dashed line in (d), indicating an approximate thickness of 25 nm for the bottom Pt layer. f) Relative height profile corresponding to the red dashed line in (d), indicating an approximate total thickness of 125 nm for the top $NbTi_xO_y$/Nb/Pt stack. The functional $NbTi_xO_y$/Nb layer has a thickness of approximately 100 nm. The atomic force microscopy images of the device were performed by the Cypher ES (Oxford Instruments) and the scanning electron microscope images were performed by the EVO 18 (Zeiss).

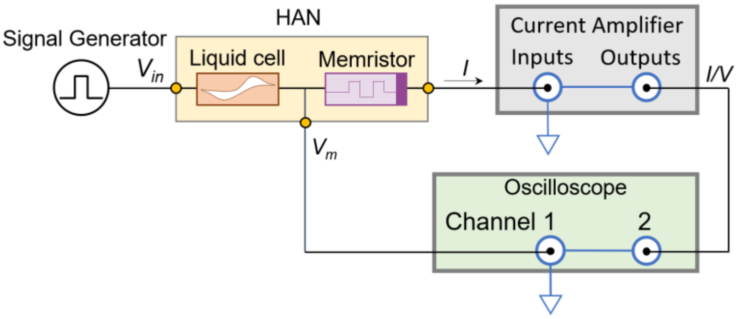

Supplementary Fig. 23. Experimental setup for simultaneous voltage and current measurements of the HAN. Input voltage V_{in} is applied by a signal generator (Tektronix AFG31052). Current (I) is measured via a current amplifier (Stanford Research SR570), whose output, proportional to current, is connected to one channel of the oscilloscope. Another oscilloscope channel is directly employed to measure the memristor voltage (V_m).

Supplementary Table 1. Energy consumption reported for typical threshold memristors. standby power is calculated as the product of current in the off-state (I_{off}) and $V_{th}/2$. ON power is determined by multiplying the threshold voltage (V_{th}) by either the on-current (I_{on}) or the compliance current (I_{cc}), both measured at V_{th}. I_{off} represents the off-current measured at $V_{th}/2$.

Functional Materials	V_{th} (V)	V_h (V)	I_{off} (A)	I_{on} (A)	I_{cc} (A)	Standby power (W)	ON power (W)
Nb/$NbTi_xO_y$ (our work)	1.2	0.9	4×10^{-5}	2×10^{-3}	/	24μ	2.4m
NbO_x[1]	0.8	0.6	1×10^{-5}	1×10^{-3}	/	4μ	800μ
VO_2[2, 3]	1.3	0.8	3×10^{-4}	/	3×10^{-3}	195μ	3.9m
Ag/HfO_x[4, 5]	0.3	0.1	1×10^{-12}	/	1×10^{-4}	150f	26μ
Ag/ Al_2O_3 /HfO_2[6]	0.036	0.01	1×10^{-7}	2×10^{-6}	/	1.8n	72n
Ta_2O_5[7]	0.7	0.3	1×10^{-11}		1×10^{-6}	3.5p	700μ
Indium–gallium–zinc–oxide (IGZO) [8]	0.7	0.3	1×10^{-10}	2×10^{-6}	/	35p	1.4μ
Ag/TiO_2[9]	0.24	0.15	1×10^{-12}	/	1×10^{-5}	132f	2.4μ
FeO_x[10]	0.6	0.1	2×10-[11]	/	1×10^{-6}	7.5p	0.6μ
Ge-Te[11]	1.1	0.4	3×10^{-8}	/	1×10^{-3}	16.5n	1.1m
Si-Te[12]	1.5	0.75	9×10^{-10}	4×10^{-4}	/	697.5p	600μ
SiO_xTe_y[13]	1.2	1	3×10^{-6}	6×10^{-5}	/	1.8μ	72μ
SnSe[14]	0.5	0.2	1×10^{-7}	/	1×10^{-5}	25n	5μ
GeSAs[15]	2.5	1.5	1×10^{-8}	1×10^{-3}	/	12.5n	2.5m
SiTe[16]	1.05	1	2×10^{-8}	/	1×10^{-3}	8.4n	1.05m
C-Te[17]	0.64	0.36	1×10^{-9}	/	5×10^{-4}	320p	320μ
GeS[18]	3.2	0.1	1×10^{-8}	/	1×10^{-2}	16n	32m
MgO/$Si_{16}Te_{45}As_{35}Ge_4$/MgO[19]	2.5	1.4	1×10^{-9}	3×10^{-4}	/	1.25n	750μ

Supplementary References

1. Duan Q., et al. Spiking neurons with spatiotemporal dynamics and gain modulation for monolithically integrated memristive neural networks. *Nat. Commun.* **11**, 3399 (2020).
2. Yuan R., et al. A calibratable sensory neuron based on epitaxial VO_2 for spike-based neuromorphic multisensory system. *Nat. Commun.* **13**, 3973 (2022).
3. Yuan R., et al. A neuromorphic physiological signal processing system based on VO_2 memristor for next-generation human-machine interface. *Nat. Commun.* **14**, 3695 (2023).
4. Midya R., et al. Anatomy of Ag/hafnia-based selectors with 10^{10} nonlinearity. *Adv Mater* **29**, (2017).
5. Hua Q., et al. A threshold switching selector based on highly ordered Ag nanodots for x-point memory applications. *Adv Sci (Weinh)* **6**, 1900024 (2019).
6. Liang H., et al. Artificial neurons based on a threshold switching memristor with ultralow threshold voltage. *ACS Appl. Electron. Mater.* **7**, 3019-3029 (2025).
7. Yao L., et al. High-speed Ta_2O_5-based threshold switching memristor for LIF neurons. *J. Appl. Phys.* **136**, 144902 (2024).
8. Mao H., et al. A spiking stochastic neuron based on stacked InGaZnO memristors. *Adv. Electron. Mater.* **8**, 2100918 (2022).
9. Jeonghwan S., Jiyong W., Prakash A., Daeseok L.& Hyunsang H. Threshold selector with high selectivity and steep slope for cross-point memory array. *IEEE Electron Device Lett.* **36**, 681-683 (2015).
10. Zhang Y., et al. Highly compact artificial memristive neuron with low energy consumption. *Small* **14**, e1802188 (2018).
11. Koo Y., Baek K.& Hwang H. Te-based amorphous binary OTS device with excellent selector characteristics for x-point memory applications. *Symp. on VLSI Tech. Dig.* 1-2 (2016).
12. Wu R., Sun Y., Zhang S., Zhao Z.& Song Z. Great potential of Si-Te ovonic threshold selector in electrical performance and scalability. *Nanomaterials.* **13**, (2023).
13. Vaziri S., et al. First fire-free, low-voltage (~1.2 V), and low off-current (~3 nA) SiO_xTe_y selectors. *2022 IEEE Symp. VLSI Technol. Circuits* (2022).
14. Qin Y., et al. Threshold switching memristor based on 2D SnSe for nociceptive and leaky-integrate and fire neuron simulation. *ACS Appl. Electron. Mater.* **6**, 4939-4947 (2024).
15. Wu R., et al. The role of arsenic in the operation of sulfur-based electrical threshold switches. *Nat. Commun.* **14**, 6095 (2023).
16. Koo Y., Lee S., Park S., Yang M.& Hwang H. Simple binary ovonic threshold switching material SiTe and its excellent selector performance for high-density memory array application. *IEEE Electron Device Lett.* **38**, 568-571 (2017).
17. Chekol S. A., et al. A C-Te-based binary OTS device exhibiting excellent performance and high thermal stability for selector application. *Nanotechnology* **29**, 345202 (2018).
18. Jia S., et al. Ultrahigh drive current and large selectivity in GeS selector. *Nat. Commun.* **11**, 4636 (2020).
19. Lee J., et al. Enhanced switching characteristics of an ovonic threshold switching device with an ultra-thin MgO interfacial layer. *IEEE Electron Device Lett.* **43**, 220-223 (2022).
